# Epidemiology of the Emergent Disease Paridae pox in an Intensively Studied Wild Bird Population

**DOI:** 10.1371/journal.pone.0038316

**Published:** 2012-11-21

**Authors:** Shelly Lachish, Becki Lawson, Andrew A. Cunningham, Ben C. Sheldon

**Affiliations:** 1 Edward Grey Institute, Department of Zoology, University of Oxford, Oxford, United Kingdom; 2 Institute of Zoology, Zoological Society of London, London, United Kingdom; University of Georgia, United States of America

## Abstract

Paridae pox, a novel avipoxvirus infection, has recently been identified as an emerging infectious disease affecting wild tit species in Great Britain. The incursion of Paridae pox to a long-term study site where populations of wild tits have been monitored in detail for several decades provided a unique opportunity to obtain information on the local-scale epidemiological characteristics of this novel infection during a disease outbreak. Using captures of >8000 individual birds, we show that, within two years of initial emergence, Paridae pox had become established within the population of great tits (*Parus major*) reaching relatively high peak prevalence (10%), but was far less prevalent (<1%) in sympatric populations of several other closely related, abundant Paridae species. Nonlinear smoothing models revealed that the temporal pattern of prevalence among great tits was characterised by within-year fluctuations indicative of seasonal forcing of infection rates, which was likely driven by multiple environmental and demographic factors. There was individual heterogeneity in the course of infection and, although recovery was possible, diseased individuals were far less likely to be recaptured than healthy individuals, suggesting a survival cost of infection. This study demonstrates the value of long-term monitoring for obtaining key epidemiological data necessary to understand disease dynamics, spread and persistence in natural populations.

## Introduction

Avian pox is a well-known viral disease of birds caused by dsDNA viruses in the genus *Avipoxvirus*, with a worldwide distribution and a wide host range [1]. Avipoxvirus infections cause proliferative ‘wart-like’ skin lesions, which are most commonly restricted to the featherless regions of the body (so-called ‘dry’ pox), but can also develop in the upper alimentary and respiratory tracts (‘wet’ or ‘diptheric’ pox) [1]. The incubation period and duration of avian poxvirus infection is variable, but affected birds with mild lesions frequently recover and this is considered to be the most common situation in endemic areas [2]. Indeed, where avian pox is endemic, the disease exists at very low prevalence and is presumed to have little impact on affected hosts [1], [3]. In naïve populations and species, however, the disease can attain a much higher prevalence and has been associated with substantial population declines [4], [5], [6].

A recent analysis of opportunistic reports of garden bird mortality and morbidity collected from 2006 to 2010 throughout Great Britain (GB) has revealed that an unusually severe form of avian pox affecting Paridae species (tits) is an emerging infectious disease in GB [7]. Although reports of avipoxvirus infections exist from multiple wild bird families and orders [1] and the disease is considered endemic in GB in non-Paridae species (e.g. common wood-pigeon *Columba palumbus*, dunnock *Prunella modularis*), avian pox was unknown within the Paridae family in GB prior to the index case observed in Sussex, England, in 2006 [7]. In addition, pox lesions in Paridae species are frequently larger and more florid than the small wart-like lesions characteristic of infections in non-Paridae species, and were considered a significant contributory factor to the cause of death of birds examined post mortem [7]. Spatio-temporal analysis of the nationwide incidence data, coupled with phylogenetic analysis of avian poxvirus core 4b gene sequences, showed that this novel ‘Paridae pox’ originated in south-east England following likely viral incursion from continental Europe and has since spread extensively throughout south and central England and into Wales [7].

Assessing the implications of novel infectious disease epizootics and accurately forecasting their epidemic trends requires a thorough understanding of disease epidemiology and dynamics in natural populations [8], [9], [10]. Monitoring epizootics in natural populations from their outset, though, is inherently difficult, and is further hindered by the financial and logistical constraints involved in regularly capturing and testing large numbers of wild animals [11]. For this reason, empirical data describing the epidemiological characteristics of emerging diseases in free-living wild animals are rare. The appearance of a novel pathogen in a long-term monitored population of wild animals, however, provides a unique opportunity to collect the crucial data necessary to aid our understanding of disease epidemiology, spread and persistence [12], [13].

In May 2009, the first case of Paridae pox in Wytham Woods, south-east England, was detected in a great tit. Wytham Woods is a long-term study site where populations of both great tits (*Parus major*) and blue tits (*Cyanistes caeruleus*) have been intensively monitored, including ringing of all breeding adults and nestlings, for more than 50 years. A substantial increase in the number of great tit cases of pox throughout 2010, coupled with the first observed cases in other species captured at this site, indicated the establishment of the disease. In this study, we take advantage of this rare opportunity to characterise the epidemiological patterns of Paridae pox among tits at a local, within-population scale. Our aims were to: (a) describe the temporal and spatial patterns in the prevalence of this viral disease from its initial appearance in the great tit population; (b) evaluate the influence of demographic factors on patterns of prevalence of the disease in great tits (host age, sex, or immigrant status: locally-born residents, versus immigrants born elsewhere); and (c) assess species-specific differences in disease prevalence.

## Methods

### Field sampling techniques

From May 2009 to November 2011, great tits were monitored in Wytham Woods near Oxford, UK (51°46′N, 1°20′W), as part of several on-going ecological studies [14], [15], [16]. This 385-ha study site is a continuous mixed semi-deciduous forest, in which approximately 1200 nestboxes are distributed at variable densities [17]. During the annual, synchronous breeding season of this species (May/June), individuals were captured between days 6 and 14 of the nestling phase, either within the nestbox by hand or using traps, or with mist nets in front of the nest entrance. Outside the breeding season, individuals were captured with mist nets set throughout the woodland in association with seed feeders. All captured individuals and all locally-born nestlings that survive to day 15 were marked with unique metal rings. Capture sessions outside the breeding season occurred on an approximately monthly basis from October 2009 to early March 2010, from October 2010 to late March 2011, and from August 2011 to November 2011. The prevalence of Paridae pox was determined as the proportion of individuals with visible pox lesions (see below) per ‘capture session’ (for the breeding seasons as a whole, and for each calendar month otherwise; with prevalence in Feb 2010, Nov 2010 and Sep 2011 determined for the calendar month plus the few trapping days conducted just outside the month assigned, see Table 1). Several other species that commonly form mixed-species foraging flocks with great were captured during field sampling (see Table 2); blue tits were captured at high rates in all capture sessions, but the remainder of species were captured predominantly outside the breeding season in mist nets; we report data from these bird species as well.

**Table 1:** Details of the capture sessions of great tits in Wytham Woods (May 2009–November 2011).

Capture Session	From	To	Trapping intensity*	‘trend’†	No. Individuals Captured	No. Diseased	Prevalence (%)	No. Diseased (Other Species)
May-09*	04/04/2009	19/06/2009	0.47	0.00	512	1	0.20	0
Oct-09	08/10/2009	28/10/2009	0.30	0.42	74	0	0.00	0
Nov-09	02/11/2009	24/11/2009	0.32	0.50	35	1	2.86	0
Feb-10	26/01/2010	04/03/2010	0.57	0.75	218	1	0.46	0
May-10*	05/05/2010	26/06/2010	0.69	1.00	554	20	3.62	0
Nov-10	20/10/2010	29/11/2010	0.65	1.50	421	37	8.79	4
Dec-10	01/12/2010	17/12/2010	0.75	1.58	144	14	9.72	0
Jan-11	02/01/2011	31/01/2011	0.41	1.67	79	3	3.80	1
Feb-11	02/02/2011	25/02/2011	0.39	1.75	93	6	6.45	1
Mar-11	03/03/2011	22/03/2011	0.47	1.83	67	2	2.99	1
May-11*	04/05/2011	05/06/2011	0.78	2.00	396	6	1.52	1
Aug-11	05/08/2011	17/08/2011	0.50	2.25	34	1	2.94	0
Sep-11	02/09/2011	4/10/2011	0.29	2.33	167	6	3.59	0
Oct-11	3/10/11	25/10/11	0.29	2.42	91	3	3.30	0
Nov-11	1/11/11	28/11/11	0.48	2.50	323	11	3.40	2

* **Trapping intensity gives the proportion of days within each capture session when trapping occurred.**

† ‘trend’ = time since initial disease occurrence (in years). Also shown are the number of individuals captured, the apparent prevalence of pox per capture session (* indicates breeding seasons), and the number of diseased individuals of other Paridae species captured during the study (see Table 2 for more details).

**Table 2:** Pox prevalence in species within the tit-nuthatch guild captured within Wytham Woods (May 2009–November 2011).

Species	Total Captures	No. Individuals Captured	No. Diseased	Pox prevalence
Great tit (*Parus major*)	3661	2076	104	5.01%
Blue tit (*Cyanistes caeruleus*)	6700	4820	3	0.06%
Coal tit (*Periparus ater)*	1013	692	4	0.58%
Marsh tit (*Poecile palustris)*	579	354	3	0.85%
Long-tailed tit (*Aegithalos caudatus)*	42	37	0	0.00%
Nuthatch (*Sitta europaea*)	107	69	0	0.00%

Values shown are the number of individuals captured, the total number of captures, and number diseased for each species, along with the species-specific prevalence of avian pox throughout the study.

Following identification of Paridae pox within our study site, strict hygiene protocols were implemented to minimise anthropogenic transmission and spread of pox. Dedicated holding bags and measuring devices for diseased birds were used. After each diseased bird was processed field workers thoroughly disinfected their hands and all equipment (including traps, and mist nets) with F10 veterinary disinfectant (www.f10biocare.co.uk). Each captured bird was carefully examined for visual signs of skin lesions. Pox lesions were identified by the presence of swellings or proliferative skin lesions, especially around the beak and eyes, legs, and on sparsely feathered parts of the wings and body. Only individuals with one or more skin lesions characteristic of pox were classified as ‘diseased’. We confirmed that this disease was caused by avian poxvirus infection by histopathologic and PCR examination of skin lesions from two great tits found dead in the woodland; see [7] for details. Previous studies have shown very good concordance between the presence of pox-like lesions and poxvirus infection; reviewed in [1]. In addition, avian pox was confirmed as the cause of the skin lesions in all suspected cases of Paridae pox in an additional 18 great tits examined as part of a larger study of the emergence and spread of this novel disease across Great Britain (via a combination of histopathology, electron microscopy and PCR) [7]. That study also reported no DNA sequence variation in the 4b core protein of the virus obtained from 20 diseased great tits [7].Hence we are confident that birds with lesions were infected with avian poxvirus and that only one virus strain was present in the population. We recognize, however, that some infected birds may have been misclassified as healthy (those that had no skin lesions or had very small lesions that were overlooked, although lesions as small as 1 mm were easily observed), and thus our estimates are of disease, rather than infection, prevalence.

### Statistical analyses

The prevalence of avian pox in species other than great tits was extremely low (see Table 2), and so statistical analyses were restricted to great tits. Since exploring only linear changes of disease prevalence through time can mask more complex temporal patterns [18], we employed a statistical approach that seeks the best linear or nonlinear fit to prevalence data. Variation in pox prevalence was examined using generalized additive modelling, essentially a generalized linear model in which a smoothed function of a covariate can be considered alongside conventional linear predictors and their interactions [19]. We incorporated a smoothed date covariate (‘trend’, calculated as the time since first observed occurrence of the disease in years based on the capture session, see Table 1) while examining associations between pox prevalence and linear functions of host sex, host age and host status (resident or immigrant), using binomial errors and a logit link. Host sex was determined based either on the presence (female) or absence (male) of a brood patch (breeding birds), or on plumage coloration (males have broad black breast stripes and glossy crowns) at other times of year [20]. During and following the autumn moult it is only possible to age newly captured individuals, based on their plumage characteristics, as either “juveniles” (aged 0–1 yr) or “older” (aged 1+ years) [20]. As a significant proportion of individuals in this study were only captured outside the breeding season (32.1%), we restricted age effects to these two age classes. We assigned ‘resident’ status to all birds that were ringed on the site as nestlings in nestboxes, and ‘immigrant’ status to all other individuals (we acknowledge that a small proportion of great tits may nest in natural cavities rather than nestboxes, and hence that a small proportion of individuals may be misclassified by this simple division).

All interactions between factors were considered in the starting model, except those involving the smoothed ‘trend’ term because too few diseased individuals were captured in most capture sessions to rigorously assess temporal interactions. The full starting model was optimized by backward stepwise elimination of non-significant terms, beginning with higher order interactions. Likelihood ratio tests (LRT) were used to determine if the removal of terms caused a significant change in model deviance and to confirm that a nonlinear smoothed function of ‘trend’ performed better than either a linear or a quadratic function of ‘trend’.

The Kuldorff spatial scan statistic implemented in SaTScan™ (http://satscan.org/) was used to test for the existence and extent of clusters of pox infection within the study site, and to identify their approximate locations and sizes [21]. Spatial analyses were restricted to the breeding season because during this period individuals are territorial and forage only in the immediate vicinity of the nest [22], so the nestbox coordinates [23] give an accurate description of an individuals' location and are likely to reflect the area where they acquire infections. Throughout the autumn and winter, birds forage and move over far greater ranges, and the locations of captured individuals (i.e. the locations of mist nets) are not likely to accurately reflect where they acquired infection. The spatial scan statistic creates a series of circular windows of variable radius around every diseased individual, each of which is set to contain from zero to a maximum proportion of the total population-at-risk (set to 25%). For each location and size of scanning window, the observed number of cases within the window is compared to the expected number of cases given by a Bernoulli model and an assumption of constant risk. A LRT is used to test the hypothesis that there is an elevated rate of infection within the window. The significance of the most likely cluster, and of secondary clusters (those that do not geographically overlap with the most likely cluster), is determined by simulated p-values using Monte Carlo methods with 999 replications, and are adjusted for the multiple testing inherent in both the many cluster locations considered, as well as the many possible collections of circles used for the scanning window [21].

## Results

### Course of infection in great tit hosts

From May 2009 to November 2011 we made 3661 captures of 2076 individual great tits, of which 104 were diagnosed as diseased (total prevalence of 5.01%; Table 2). Diseased individuals possessed pox lesions ranging in size from 1 mm to 21 mm in diameter (median = 6.0 mm). Only 14 diseased individuals were captured with pox lesions on two separate occasions; none more often than this (Table 3). These individuals were mostly captured a few days apart with little change in the size of their lesions, but three birds were captured at 17, 22 and 36 days apart, the latter individual displaying substantial growth in lesion size over this period (>10 mm growth, constituting a five-fold increase in the size of one lesion; Table 3). Recovery from pox was apparent (in one case within as little as one week), with 15 diseased individuals recaptured at a later date without pox lesions (Table 4). Recovered individuals formed an even mix of males and females, of both age classes, possessed lesions varying in size and location on the body and, in all but one case, displayed no visible scarring at the site of the original lesion. Only one of the 15 diseased individuals that recovered from pox infection was ever subsequently recaptured again (one year later, apparently still pox free). Nevertheless, the majority of diseased individuals (77.8%; 81/104) were never recaptured. Indeed the return rate (i.e. the proportion of marked individuals ever recaptured) of diseased individuals was far lower than for healthy individuals. For example, healthy individuals captured in May 2010 were 3.4 times more likely to be recaptured at least once after marking than were diseased individuals (odds ratio 95% CI = 1.03–12.99; Fisher's exact test P = 0.045), with stronger results for the November 2010 capture ‘cohort’ (healthy individuals were 4.61 times more likely to be recaptured than diseased individuals; odds ratio 95% CI = 1.59–18.32, P = 0.001).

**Table 3:** Details of birds captured twice with pox lesions.

Recapture Interval (days)	Change in size (mm) of lesion(s)
2	no change
2	no change
2	+1
3	+1
3	NA
3	no change
3	NA
3	no change
4	no change
4	NA
8	+1
17	+2
22	−1
36	+12 & +7

Values shown are the change in the size of lesions between capture occasions for individuals captured twice with disease.

**Table 4:** Details of birds that recovered from pox.

Recovery Interval (days)	Size (mm)/location of lesion(s)
7	3/head
15	2/eye
54	3/foot
82	5/beak
89	13/eye
90	3/foot
154	6/thigh
154	5/eye
160	2/beak
160	2/eye
180	7/eye
359	8/eye
359	10 & 7.5/head & wing
365†	“large”/head
556	3/foot

Values shown are the intervals between the last disease capture and the first healthy capture of each recovered individual, along with the size and location of the original pox lesions.

† This was the only recovered individual to be subsequently recaptured.

### Species-specific differences in disease prevalence

Six species within the tit-nuthatch guild were captured during this study (Table 2). Pox prevalence was substantially higher in great tits, than in any other species captured and examined (Table 2). Particularly striking is the almost one hundred-fold difference in pox prevalence in the great tit population (5.01%) compared with the blue tit population (0.06%), as this species was both abundant and frequently recaptured during the study (Table 2). None of the diseased blue tits, coal tits or marsh tits were recaptured at a later date.

### Temporal and demographic trends in apparent prevalence

**Figure 1:**
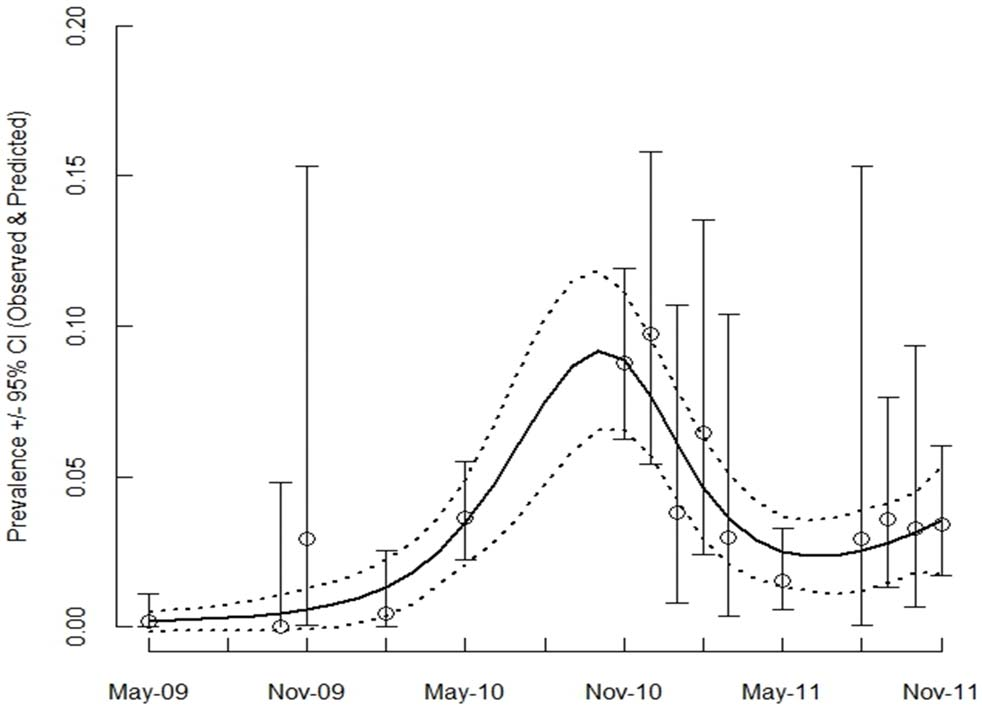
Observed prevalence of pox in great tits (with 95% exact binomial confidence intervals). The solid line represents the fit of the predicted values from the final generalised additive model (see Table 5). Dotted lines show 95% CI of the predicted prevalence estimates.

**Figure 2:**
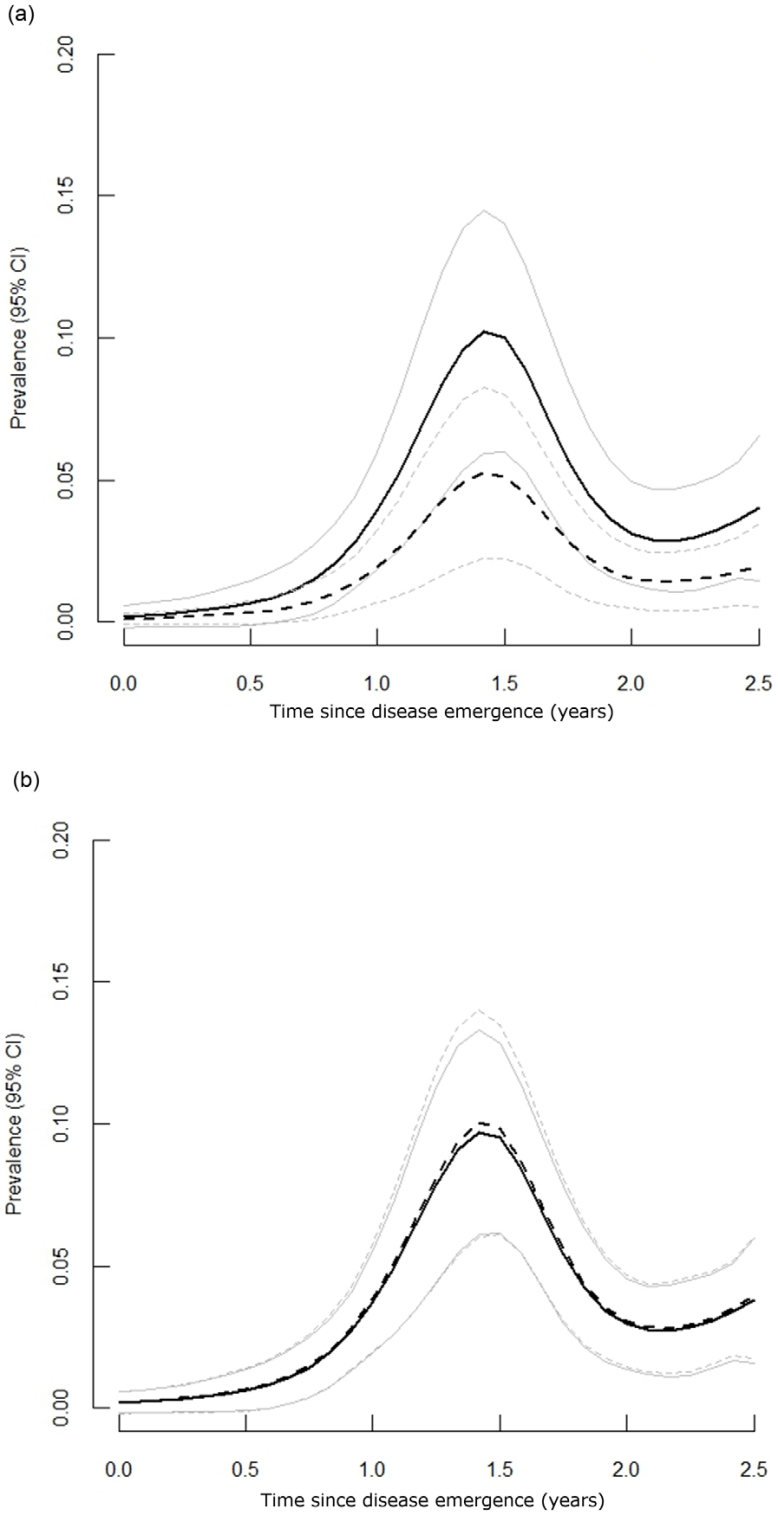
Predicted pox prevalence in residents (solid lines) and immigrants (dashed lines) according to host age. (a) adults (1+ years of age) and (b) juveniles (<1 year). Light grey solid and dashed lines are the 95% CI of the estimates of the predicted prevalence for residents and immigrants respectively.

A nonlinear smoothed function of ‘trend’ (time since initial sighting of disease) was retained as the most suitable temporal predictor of Paridae pox prevalence (Table 5). The model including a nonlinear function of ‘trend’ was preferred over either a linear or a quadratic function of trend (LRT *s*(‘trend’) vs. ‘trend’: Δdeviance = 62.49, Δdf = 3.35, P<0.001; *s*(‘trend’) vs. ‘trend’+‘trend^2^’: Δdeviance = 21.661, Δdf = 2.35, P<0.001), and provided a better fit to the data (*R* ^2^ for the nonlinear model = 0.75, the linear model = 0.32, and the quadratic model = 0.54). The predicted pox prevalence over our sampling period (predictions generated from the final model) provided a generally good fit to our observed prevalence data and showed that prevalence remained relatively low for the year following the initial disease outbreak, but then increased to peak in the autumn and early winter of 2010 (Fig. 1). Over spring and summer of 2011 prevalence declined, but increased again in autumn 2011 (although not to the same extent), indicative of seasonality in poxvirus infection rates (Fig. 1). Also, the majority of the avian pox cases in other tit species were observed during these late autumn peak periods (Table 1), supporting the idea of a higher force of infection at this time of year relative to spring and summer. An interaction between host age-class and host status was also retained in the final model (at near significance P = 0.055, Table 5). For older individuals (1+ years of age) pox prevalence was higher among residents than immigrants (Fig. 2a), but this was not the case for juveniles (0–1 years of age; Fig. 2b). Overall, prevalence in older residents was approximately twice as high (4.74%; CI = 3.11–6.86) as in older immigrants (2.17%; CI = 1.16–3.67).

**Table 5:** Results of models examining the influence of temporal/demographic factors on variation in pox prevalence.

Factors	Parameter Estimate	Z/χ^2^	Wald's test (P)	ΔDev/Δdf	LRT (P)
**smoothed ‘trend’**	**estimated df = 4.461**	**26.73**	**<0.0001**	**78.862/4.56**	**<0.0001**
**ageclass (older)**	**−0.677±0.331**	**−2.046**	**0.041**	–	–
**status (resident)**	**−0.036±0.243**	**−0.148**	**0.882**	–	–
ageclass*status	**0.795±0.424**	**1.877**	**0.060**	**3.72/1**	**0.055**
sex (M)	−0.078±0.197	−0.396	0.692	0.220/1	0.642
status*sex	0.104±0.395	0.263	0.792	0.063/1	0.801
ageclass*sex	−0.278±0.416	−0.669	0.503	0.467/1	0.495
ageclass*sex*status	0.750±0.860	0.872	0.383	0.778/1	0.379

Values shown are the Z statistics (or **χ^2^** statistics for the smoothed term) and Wald's test P-values for coefficients, along with results of Likelihood ratio tests (LRT) (change in deviance: ΔDev, change in degrees of freedom: Δdf) comparing models with and without the specific term. Factors in bold were retained in the final model.

### Spatial patterns of poxvirus infection

**Figure 3:**
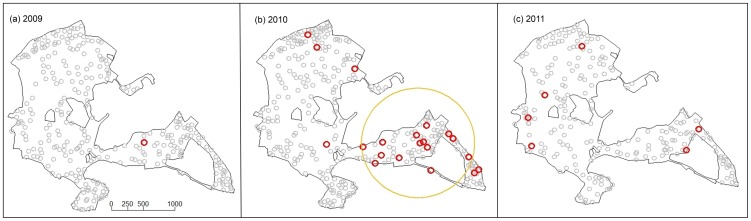
Locations of diseased (red circles) and healthy (grey circles) great tits captured in Wytham Woods. Spatial analyses were conducted for the (a) 2009, (b) 2010, and (c) 2011 breeding seasons. The location and spatial extent of the single statistically significant pox cluster identified in 2010 by the SatScan spatial cluster analysis is shown by an orange circle (log-likelihood ratio = 10.55, relative risk = 7.93; P = 0.01).

As expected, based on the north-westerly spread of Paridae pox from an origin in Sussex, south-east England [7], this disease first appeared in the south-east of our study site (Fig. 3a). Throughout the 2010 breeding season, cases remained clustered in the south-east region of the study site, where the relative risk of pox infection was estimated to be approximately eight times greater than in the rest of the woodland (Fig. 3b). By the following breeding season, however, no disease clustering was evident, with pox cases randomly distributed throughout the whole study site (Fig. 3c).

## Discussion

Paridae pox is a novel, emerging infectious disease of wild tit species across Great Britain [7]. Here, we have characterised the epidemiology of the emergence and establishment of this disease at a local, within-population, scale. We have shown that there are striking differences in the prevalence of pox infections among closely-related, sympatric Paridae species with very similar ecologies, that temporal variation in pox prevalence among great tits displays regular fluctuations indicative of seasonality in infection rates and, that while recovery is possible, diseased individuals are far less likely to be recaptured than are healthy individuals.

The prevalence of Paridae pox in the great tit population (maximum 10%, mean 5%) was higher than that reported for avian pox in wild birds where the disease is endemic (0.5–1.5%) [1]. This is consistent with Paridae pox being a novel disease in a naïve host population. The maximum prevalence of pox recorded, though, was far lower than the prevalence reported for avian pox in some other naïve populations, particularly those on islands [3], [6], [24]. Island-endemic species, however, are expected to be more vulnerable to novel disease threats owing to their evolution in the face of a depauperate pathogen complement [25]. The prevalence of avian pox in great tits in Wytham Woods was much higher than in any of the other captured species within our study site (although prior reports of avian pox exist for all these species, with the exception of long-tailed tits) [7]. In particular, pox prevalence was much higher among great tits than among the closely-related blue tits, which are both abundant and frequently captured within our study site. As great and blue tits are sympatric and ecologically similar, for example often forming mixed-species flocks [17], it seems unlikely that observed differences in species-specific prevalence can be attributed to differential exposures of these species to the pathogen. Rather, it suggests that Paridae species either differ in their susceptibility to infection (a commonly reported finding in other studies) [3], [6], [26], [27], or that the virulence of this pathogen differs between host species [28]. It is also possible, however, that such species-specific patterns of disease prevalence might simply be a consequence of differences in the detectability of diseased individuals of different species [29]. Experimental infection studies will be necessary to distinguish between these alternative possibilities.

Our analyses suggest that, following the initial period of disease establishment in the great tit population, the prevalence of avian pox in great tits began to display regular temporal oscillations, indicative of seasonal forcing of infection rates. Theoretical models show that the strength and mechanisms of seasonality can alter the spread and persistence of infectious diseases, with seasonal forcing a crucial determinant of the longer-term dynamics and fluctuations of host–parasite systems [30]. This has been supported by field observations for several wildlife infections, including both directly-transmitted and vector-transmitted parasites [31], [32], [33]. In this study, the prevalence of pox in great tits was predicted to be highest throughout autumn and early winter, which is consistent with the temporal pattern of Paridae pox cases reported nationwide [7] and with the findings of many other studies of avian pox in wild birds [1], [34]. This was supported by our finding that the majority of pox cases in other species were also observed at this time of year. The higher prevalence of Paridae pox in autumn/early winter could be driven by the increased abundance of vectors in the warmer late summer and early autumn months enhancing virus transmission rates. Mosquitoes are considered a particularly important biting insect vector for avian poxvirus transmission [1]. Several studies have found strong concordance between pox prevalence in wild birds and the abundance or distribution of mosquito vectors [26], [35], or have shown strong correlations between pox prevalence and the climatic conditions preferred by mosquitoes (higher temperatures and rainfall) [36]. Preliminary investigations into mosquito ecology at our study site have found 14 species present within Wytham Woods. All of these species are apparently abundant during the summer months (June through August) but begin to decline in numbers in September (R. Alves, unpub. data), before the apparent yearly peaks in Paridae pox prevalence in this system. This temporal lag between peak mosquito abundance and peak pox prevalence might be due to a latent period between infection and disease for this poxvirus strain [37], but also could reflect a weaker role for mosquitoes in pathogen transmission in this system. A better understanding of the temporal variation in mosquito abundance and distribution within our study site, as well as the ecology and abundance of other vectors, would help to elucidate the role of vector abundance in driving seasonality, and also the magnitude of seasonal peaks in Paridae pox prevalence.

Alternatively, the seasonal increase of pox in autumn could be facilitated by the influx of immunologically naïve juveniles that follows the synchronous spring breeding period of this species; this acts to increase both host population density and the abundance of susceptible individuals in the population [30]. According to this hypothesis, the prevalence of avian pox should be higher in juveniles than in adults, a finding which is supported by many [1], [34], [36] but not all [38], [39] avian pox studies. In our study, we did not find a clear difference in Paridae pox prevalence between juveniles and older birds, but we did find a lower overall prevalence of pox in older immigrant birds. Rather than signify age-specific differences in susceptibility to infection, however, this result is more readily explained in terms of differences in the relative exposure times for the different groups. Older residents that were ringed on our study site would have been exposed to the novel pathogen for longer on average than the more-recently arrived older immigrants, which were likely born elsewhere (presumably where disease was absent). Alternatively, but perhaps less likely, older immigrants might have already been exposed/diseased and recovered before moving into Wytham Woods. The difference in exposure times (and consequently prevalence) between resident and immigrant juveniles, however, would not be as extreme, as both would only have been in the population for a matter of months. Whether this pattern persists as Paridae pox becomes established in surrounding populations remains to be seen. Clearly, though, a thorough understanding of the role of the annual cohort of naïve juveniles in the seasonal dynamics of Paridae pox will require longer-term data on age-specific prevalence.

Post-breeding flocking behaviour also may facilitate transmission by increasing conspecific and interspecific contact rates. Epidemiological models of *Mycoplasma gallisepticum* (MG) dynamics in house finches require the inclusion of both winter aggregation and pulsed breeding events to generate the observed bi-annual peaks in prevalence [40]. Similarly, the congregation of birds at feeding stations throughout the colder months, when anthropogenic provisioning of wild birds in garden habitats in Great Britain becomes more frequent [41] and natural food resources more rare, could increase inter- and intra-specific contacts and facilitate avian poxvirus transmission. The aggregation of birds at artificial seed feeders is thought to have contributed to pathogen spread in the house finch-MG system [42]. A more-thorough investigation of the strength and regularity of the seasonal peaks in Paridae pox occurrence, and the relative importance of the numerous potential factors that might contribute to driving seasonal variation in this system, is clearly warranted, but will require additional time series data.

The results of this study reveal that the course of Paridae pox disease is highly variable among individual hosts. We found that some individuals could sustain the disease for relatively long periods of time (>1 month) with either no change or substantial growth in lesion size occurring during that time. Similar variability in the course of avian pox has been reported in other studies [26],[37]. In addition, the probability of recovery from disease in this study did not seem dependent on the size or location of lesions, as is commonly assumed [1], [37]. These patterns suggest that individual differences in immune system function are likely to exert significant control over the severity and duration of disease. Recovery from pox depends on cell-mediated immunity, and immunity to avipoxvirus in captive birds is known to be highly variable [37]. Very little is known, however, about the development or duration of immunity to poxvirus infections in wild birds. Given the importance of variation in individual and population immunity for predicting disease dynamics and persistence [40], this seems an urgent and fruitful area for further research.

While Paridae pox might not be invariably fatal, the disease may nonetheless increase the vulnerability of individuals to other causes of mortality, such as predation, since lesions can seriously impair an individual's vision, as well as its flight and feeding abilities [1], [3]. The majority of diseased individuals in this study were not recaptured, and diseased individuals had far lower return (recapture) rates than healthy birds. These findings suggest a survival cost of the disease, but need to be interpreted with caution because return rates confound both survival and detection, and detection rates themselves may differ depending on disease state [29]. An accurate assessment of the effect of Paridae pox on the survival rates of great tits will require the application of more rigorous mark-recapture statistical methods that overcome these limitations, and is the focus of our current research. Indeed, with this novel disease now well established within the Wytham Woods population of great tits, and continuing to spread across Great Britain [7], it is critical that we quantify the impact of the disease on both host survival and reproductive success in order to assess the consequence of this emerging infectious disease for the long-term viability of both the Wytham Woods and the national great tit populations. This information will also be vital for implementing effective disease interventions or management actions should they be required.
